# Food insecurity and sleep health by race/ethnicity in the United States

**DOI:** 10.1017/jns.2023.18

**Published:** 2023-05-18

**Authors:** Dana M. Alhasan, Nyree M. Riley, W. Braxton Jackson II, Chandra L. Jackson

**Affiliations:** 1 Epidemiology Branch, National Institute of Environmental Health Sciences, National Institutes of Health, Department of Health and Human Services, Research Triangle Park, NC, USA; 2 Social & Scientific Systems, Inc., a DLH Holdings Company, Durham, NC, USA; 3 Intramural Program, National Institute on Minority Health and Health Disparities, National Institutes of Health, Department of Health and Human Services, Bethesda, MD, USA

**Keywords:** African Americans, Food assistance, Food insecurity, Hispanic Americans, Minority groups, Sleep, Sleep initiation and maintenance disorders, Socio-economic factors, CI, confidence interval, NH, non-Hispanic, PR, prevalence ratio, se, standard error, SES, socio-economic status, US, United States

## Abstract

Food insecurity, poised to increase with burgeoning concerns related to climate change, may influence sleep, yet few studies examined the food security-sleep association among racially/ethnically diverse populations with multiple sleep dimensions. We determined overall and racial/ethnic-specific associations between food security and sleep health. Using National Health Interview Survey data, we categorised food security as very low, low, marginal and high. Sleep duration was categorised as very short, short, recommended and long. Sleep disturbances included trouble falling/staying asleep, insomnia symptoms, waking up feeling unrested and using sleep medication (all ≥3 d/times in the previous week). Adjusting for socio-demographic characteristics and other confounders, we used Poisson regression with robust variance to estimate prevalence ratios (PRs) and 95 % confidence intervals (95 % CIs) for sleep dimensions by food security. Among 177 435 participants, the mean age of 47⋅2 ± 0⋅1 years, 52⋅0 % were women, and 68⋅4 % were non-Hispanic (NH)-White. A higher percent of NH-Black (7⋅9 %) and Hispanic/Latinx (5⋅1 %) lived in very low food security households than NH-White (3⋅1 %) participants. Very low *v.* high food security was associated with a higher prevalence of very short (PR = 2⋅61 [95 % CI 2⋅44–2⋅80]) sleep duration as well as trouble falling asleep (PR = 2⋅21 [95 % CI 2⋅12–2⋅30]). Very low *v.* high food security was associated with a higher prevalence of very short sleep duration among Asian (PR = 3⋅64 [95 % CI 2⋅67–4⋅97]) and NH-White (PR = 2⋅73 [95 % CI 2⋅50–2⋅99]) participants compared with NH-Black (PR = 2⋅03 [95 % CI 1⋅80–2⋅31]) and Hispanic/Latinx (PR = 2⋅65 [95 % CI 2⋅30–3⋅07]) participants. Food insecurity was associated with poorer sleep in a racially/ethnically diverse US sample.

## Introduction

Food insecurity – a major public health concern – is defined as limited ability or lack of access for households to provide sufficient food for an active, healthy life among all household members^(1)^. In 2020, it was estimated that 38 million people in the United States (US) lived in food insecure households where 9⋅4 million adults lived in very low food secure households^(2)^. When one or more household members lacked sufficient financial resources, disruptions to their eating pattern occurred thus reducing food intake^(2)^. Additionally, food insecurity disproportionately impacts minoritised racial/ethnic adults in the US including Black/African Americans, hereafter non-Hispanic (NH)-Black, and Hispanic/Latinx, compared with NH-White adults^(2)^. Based on the US Department of Agriculture (USDA), NH-Black (21⋅7 %) and Hispanic/Latinx (17⋅2 %) households have higher food insecurity prevalence compared with NH-White households (7⋅1 %)^(2)^. Similarly, food insecurity differentially impacts women where food insecure women compared with men are less likely to meet the recommended dietary intake^(3)^.

Food insecurity negatively impacts the health of household members where the lack of a nutritious diet is associated with chronic illnesses, such as type II diabetes mellitus, chronic kidney disease, cardiovascular diseases^(4–6)^, as well as sleep^(7)^. The uncertainty of when one's next meal will be or even being forced to make decisions between paying a bill (e.g. rent) *v*. buying food heightens psychological distress thus activating the sympathoadrenal medullary system and hypothalamic–pituitary–adrenal (HPA) axis, which are both implicated in poor sleep health^(8)^. Psychological distress has been shown to impact sleep^(9)^. Additionally, malnutrition hinders the body from appropriately assimilating nutrients and subsequently leads to immune deficiency potentially placing individuals at higher risk for recurrent infections and chronic inflammation^(10)^ that in turn may alter sleep^(11)^. Therefore, there are both indirect and direct pathways by which sleep may be affected by food security status. Since sleep is a modifiable health behaviour that has been identified as a risk factor for chronic illnesses, it may be fundamental to addressing racial/ethnic inequities^(12)^. Furthermore, a review has highlighted that minoritised racial/ethnic groups are more likely to experience shorter sleep^(13)^. It has also been found that women are more likely to be diagnosed with insomnia or report difficulty falling and staying asleep^(14)^.

Predominantly NH-White *v*. NH-Black neighbourhoods have, on average, four times as many grocery stores^(15)^. Fewer grocery stores coupled with other limited resources (e.g. limited transportation; less high-paying jobs) lessen the ability to access viable quality and quantity food options, worsen food security status and subsequently sleep^(16,17)^. These disparities are likely due to structural racism where policies translate and equate to limited material resources in minoritised racial/ethnic neighbourhoods^(18)^. Therefore, it is likely that lower food security status may be related to poorer sleep health, including more sleep disturbances, among minoritised populations. Food insecurity and sleep are both important modifiable factors making it important to investigate the food security status–sleep health relationship among racially/ethnically diverse US adults. Additionally, it is important to know who is most impacted by food insecurity and poor sleep health to implement intervention strategies aimed at certain subgroups.

Despite its importance, few studies have been conducted in the US^(19)^. Among these few studies, most do not consider racial/ethnic differences in food security status and sleep health^(17,20,21)^ and even fewer include NH-Asians^(22–24)^. Furthermore, some studies do not assess multiple domains of food security status^(22,25)^. Prior studies of food insecurity and sleep health have been limited by their usage of small, non-diverse populations, lack of investigation by race/ethnicity^(17)^ and sex/gender despite potential unique experiences, absence of multiple sleep dimensions beyond duration^(24)^, and lack of a usage of a validated food security scale^(21)^. To overcome these research gaps, we sought to assess the prevalence of food security status and sleep health by race/ethnicity, to determine the association between food security status and sleep health, and to assess potential racial/ethnic variation in this association within a population of NH-White, NH-Black, Hispanic/Latinx and NH-Asian US women and men. We hypothesised that the prevalence of food security status and poorer sleep health will be higher among minoritised compared with NH-White women and men. We also hypothesised that participants with very low, low and marginal *v*. high food security status will have a higher prevalence of shorter sleep duration and more sleep disturbances and that this will be stronger among minoritised compared with NH-White women and men. We further hypothesised that minoritised racial/ethnic groups with very low, low, marginal and high food security status will have a higher prevalence of poorer sleep quality *v.* NH-White adults with high food security status.

## Methods

### Data source: National Health Interview Survey

We collected participant data from the National Health Interview Study (NHIS). The NHIS – a series of annual, cross-sectional, household surveys – is conducted via computer-assisted in-person interviews among non-institutionalised US citizen adults by trained interviewers. To obtain a nationally representative sample, the NHIS utilises a three-stage stratified cluster probability sampling design. All publicly available NHIS data has been extensively reviewed by the National Center for Health Statistics’ Disclosure Review Board to protect the confidentiality of survey participants, and a detailed description of NHIS procedures has been previously published^(26)^. We used survey data from 2013 to 2018 to increase sample size and decrease likelihood of non-representative results stemming from data collected in a single year. The national prevalence of food insecurity ranged from 11% to 14 % during this timeframe^(27)^. The final response rate for sampled adults was 56⋅1 % (range: 61⋅2 % (2013) – 53⋅1 % (2018)). Furthermore, each study participant provided informed consent to the NHIS, and the National Institute of Environmental Health Sciences’ Institutional Review Board waived approval for this study as de-identified, publicly available data are not classified as human subjects’ research.

### Study population

Among 190 113 participants ≥18 years old, those with missing data regarding food security status (*n* 44), sleep duration (*n* 6126), sleep disturbances (*n* 1643) and race/ethnicity (*n* 3464) were excluded, as well as those who self-identified as Native American and multiple additional racial/ethnic groups (*n* 1401) due to a small sample size. These exclusions resulted in a final analytic sample of 177 435 participants (Supplementary Figure S1).

### Exposure assessment: food security status

Food security status was measured via the routinely used tool to monitor food security: the US Household Food Security Survey Module (HFSSM) based on the USDA Economic Research Service recommendations^(28)^. This screening tool was adopted from the 18-item household module to have less respondent burden and avoided asking questions about children's food security. It consisted of ten questions about food availability and consumption in the past 30 d where participants answered yes *v*. no (Supplementary Table S1). For example, participants answered questions about their households if they ‘worried whether food would run out before we got money to buy more’; ‘the food we bought just didn't last, and didn't have money to get more’; ‘we couldn't afford to eat balanced meals’ and ‘you or other adults in family ever cut the size of your meals or skip meals because there wasn't enough money for food?’. Among those who answered ‘yes’, participants answered often true, sometimes true, *v*. never true (3 d or more *v*. <3 d). Affirmative responses were summed, ranged from 0 to 10, and categorised as high food security (0), marginal food security (1–2), low food security (3–5) and very low food security (6–10) based on USDA coding^(29)^.

### Outcome assessment: sleep duration and sleep disturbances

Participants answered the following question regarding sleep duration, ‘On average, how many hours of sleep do you get in a 24 h period?’. Responses were reported in hours using whole numbers and rounded up values equal to or greater than 30 min to the nearest hour and rounded down values less than 30 min to the nearest hours. Based on the recommendation of the National Sleep Foundation categories^(30)^ that have been previously validated^(31)^, the following sleep duration categories were used: very short sleep (<6 h), short sleep (<7 h), recommended sleep (7–9 h) and long sleep (≥9 h). Categories of very short and short were not mutually exclusive.

Participants answered the following four questions regarding sleep disturbances: (1) ‘In the past week, how many times did you have trouble falling asleep?’; (2) ‘In the past week, how many times did you have trouble staying asleep?’; (3) ‘In the past week, how many times did you take medication to help you fall asleep or stay asleep?’ and (4) ‘In the past week, how many days did you wake up feeling rested?’ A response of 3 d/times or more a week indicated a self-reported sleep disturbance: trouble falling asleep, trouble staying asleep, sleep medication use and waking up feeling unrested. We also defined insomnia symptoms as having trouble falling or staying asleep 3 or more nights per week *v*. less than 3 nights per week in efforts to capture people with moderate to severe rather than occasional insomnia symptoms.

### Potential confounders

Confounders were based on *a priori* literature. Since the NHIS uses self-reported data for sex where it is unclear how participants perceived this question, we combined sex and gender, hereafter, sex/gender (women/men). We included race (White, African American or Black, Hispanic/Latinx or Asian) and ethnicity (non-Hispanic/Latinx or Hispanic/Latinx), which we combined since these categories are mutually exclusive; therefore, race/ethnicity consisted of NH-White, NH-Black, Hispanic/Latinx and NH-Asian. Other potential socio-demographic confounders included age (18–30, 31–50 or ≥50), educational attainment (<high school, high school, some college or ≥college graduate), annual household income (<$35 000, $35 000–$74 999 and ≥$75 000), employment status (employed/in labour force or unemployed/not in labour force), occupational class (professional/management, support services or labourers), marital status (married/living with partners/co-habitating, divorced/widowed or single/no live-in partner) and region of residence (Northeast, Midwest, South and West). We did not consider health behaviours, such as smoking status, or clinical characteristics, such as severe psychological distress, because they are considered potential mediators of the relationship between food security status and sleep health^(21,32)^.

### Potential modifiers: sex/gender and race/ethnicity

Sex/gender was assessed in a binary manner and dichotomised between women *v*. men. Race/ethnicity was categorised as NH-White alone, NH-Black alone, Hispanic/Latinx of any race and NH-Asian.

### Statistical analyses

Descriptive statistics were computed; continuous variables were presented as means and standard errors (se), and categorical variables were presented as weighted percentages after applying direct age standardisation using the 2010 US Census as the standard population. We compared the four levels of food security status (very low, low, marginal and high) across socio-demographic, health behaviours and clinical characteristics for all participants.

To test associations between food security and sleep dimensions, we used Poisson regression with robust variance as a valid approach to directly estimate prevalence ratios (PRs)^(33)^ and 95 % confidence intervals (CIs) of very low, low, marginal *v*. high food security for each sleep dimension overall and by race/ethnicity and by sex/gender. This model with adjusted variances is used for either count or binary data for cross-sectional (or longitudinal) studies to provide accurate point and interval estimates, correct standard errors when over or under-dispersion is observed and directly estimate PRs^(33)^. Unlike odds ratios estimated from logistic regression models, PRs do not overestimate associations with outcomes of high prevalence (e.g. poor sleep health). PRs are also easier to interpret and communicate^(33)^.

We also compared very low, low, marginal and high food security among minoritised racial/ethnic groups to NH-White participants with high food security. The overall model was adjusted for the following confounders: age, race/ethnicity, sex/gender, educational attainment, annual household income, employment status, occupational class, region of residence, marital/co-habitating status and employment status. To test for differences by race/ethnicity and by sex/gender and food security status, we added respective interaction terms (e.g. food security status*race/ethnicity; food security status*sex/gender) to the overall model. Analyses were conducted using SAS version 9.4 for Windows (Cary, North Carolina), and a two-sided *P*-value of 0⋅05 was used to determine statistical significance.

## Results

### Study population characteristics

Among 177 435 participants, the majority (82⋅9 %) lived in households with high food security status followed by marginal (6⋅8 %), low (5⋅7 %) and very low (4⋅5 %) (Table 1). The mean age was 47⋅2 ± 0⋅09 years. Approximately 52⋅0 % were women and 68⋅4 % self-identified as NH-White, 11⋅4 % as NH-Black, 14⋅6 % as Hispanic/Latinx and 5⋅6 % as NH-Asian. NH-Black participants lived in higher percentage of households with very low food security status (23⋅9 %) compared with high food security status (9⋅7 %), while NH-White and NH-Asian participants lived in higher percentage of households with high food security status (71⋅3 and 6⋅0 %, respectively) compared to a very low food security status (54⋅4 and 2⋅4 %, respectively) (Table 1).

**Table 1.** Age-standardised socio-demographic, health behaviour and clinical characteristics between very low, low, marginal and high food security, National Health Interview Survey, 2013–2018 (*N* 177 435)^a^

	Food security status				
	Very low *n* 7998 (4⋅5 %)	Low *n* 10 155 (5⋅7 %)	Marginal *n* 12 132 (6⋅8 %)	High *n* 147 150 (82⋅9 %)	Overall *n* 177 435 (100 %)
Socio-demographic					
Age, mean (se), years	43⋅4 (0⋅25)	43⋅0 (0⋅24)	42⋅9 (0⋅22)	48⋅0 (0⋅10)	47⋅2 (0⋅09)
18–30	16⋅6 %	18⋅2 %	18⋅2 %	16⋅1 %	16⋅4 %
31–50	23⋅0 %	21⋅5 %	21⋅5 %	23⋅5 %	23⋅2 %
≥50	60⋅3 %	60⋅3 %	60⋅3 %	60⋅3 %	60⋅3 %
Sex/gender					
Women	56⋅6 %	56⋅6 %	56⋅6 %	51⋅2 %	52⋅02 %
Race/ethnicity					
NH-White	54⋅4 %	47⋅6 %	53⋅0 %	71⋅3 %	68⋅4 %
NH-Black	23⋅9 %	22⋅6 %	19⋅2 %	9⋅7 %	11⋅4 %
Hispanic/Latinx	19⋅3 %	26⋅1 %	23⋅3 %	13⋅1 %	14⋅6 %
NH-Asian	2⋅4 %	3⋅7 %	4⋅5 %	6⋅0 %	5⋅6 %
Educational attainment					
<High school	24⋅5 %	25⋅2 %	20⋅5 %	8⋅3 %	10⋅4 %
High school graduate	34⋅9 %	36⋅3 %	34⋅8 %	25⋅9 %	27⋅5 %
Some college	31⋅6 %	28⋅2 %	29⋅9 %	30⋅1 %	30⋅1 %
≥College	8⋅9 %	10⋅4 %	14⋅9 %	35⋅7 %	32⋅0 %
Annual household income					
<$35 000	75⋅4 %	67⋅2 %	54⋅8 %	22⋅7 %	29⋅0 %
$35 000–$74 999	20⋅1 %	25⋅4 %	31⋅0 %	30⋅7 %	30⋅3 %
≥$75 000	4⋅5 %	7⋅4 %	14⋅2 %	46⋅6 %	40⋅7 %
Unemployed/not in labour force	65⋅1 %	58⋅3 %	51⋅2 %	37⋅9 %	41⋅0 %
Occupation class					
Professional/management	7⋅5 %	7⋅9 %	10⋅1 %	23⋅7 %	21⋅4
Support services	39⋅9 %	40⋅3 %	41⋅6 %	45⋅8 %	45⋅1
Labourers	52⋅6 %	51⋅8 %	48⋅2 %	30⋅6 %	33⋅4
Marital status					
Married/living with partner/co-habited	36⋅4 %	45⋅4 %	51⋅4 %	63⋅7 %	61⋅1 %
Divorced/widowed	38⋅0 %	31⋅9 %	27⋅5 %	19⋅1 %	20⋅8 %
Single/no live-in partner	25⋅6 %	22⋅7 %	21⋅1 %	17⋅2 %	18⋅1 %
Region of residence					
Northeast	15⋅3 %	16⋅1 %	18⋅0 %	18⋅4 %	18⋅2 %
Midwest	21⋅0 %	19⋅2 %	21⋅2 %	22⋅7 %	22⋅4 %
South	43⋅0 %	43⋅9 %	39⋅8 %	35⋅9 %	36⋅7 %
West	20⋅7 %	20⋅8 %	21⋅1 %	23⋅0 %	22⋅7 %
**Health behaviours**	**Very Low**	**Low**	**Marginal**	**High**	**Overall**
Smoking status					
Never/quit >12 months prior	61⋅2 %	72⋅7 %	76⋅0 %	86⋅0 %	83⋅6 %
Former	2⋅3 %	1⋅4 %	2⋅0 %	1⋅3 %	1⋅3 %
Current	36⋅6 %	25⋅8 %	22⋅1 %	12⋅8 %	15⋅0 %
Alcohol consumption					
Never	23⋅3 %	26⋅6 %	24⋅0 %	18⋅6 %	19⋅4 %
Former	24⋅9 %	21⋅6 %	21⋅0 %	14⋅1 %	15⋅2 %
Current	51⋅9 %	51⋅8 %	55⋅0 %	67⋅3 %	65⋅3 %
Leisure-time physical activity					
Never/unable	50⋅2 %	47⋅9 %	42⋅8 %	29⋅7 %	32⋅2 %
Does not meet PA guidelines	18⋅3 %	19⋅1 %	19⋅5 %	18⋅9 %	19⋅0 %
Meets PA guidelines^b^	31⋅5 %	33⋅0 %	37⋅6 %	51⋅4 %	48⋅8 %
Sleep duration					
<6 h	23⋅6 %	15⋅5 %	13⋅4 %	7⋅7 %	9⋅1 %
<7 h	50⋅4 %	41⋅0 %	38⋅2 %	29⋅7 %	31⋅5 %
7–9 h	42⋅7 %	53⋅1 %	55⋅9 %	66⋅7 %	64⋅4 %
>9 h	6⋅8 %	5⋅9 %	5⋅9 %	3⋅6 %	4⋅0 %
Trouble falling asleep (≥3 nights)	46⋅8 %	34⋅6 %	28⋅2 %	17⋅4 %	20⋅1 %
Trouble staying asleep (≥3 nights)	50⋅9 %	38⋅3 %	33⋅4 %	25⋅2 %	27⋅4 %
Insomnia symptoms^c^	59⋅3 %	46⋅3 %	41⋅2 %	30⋅5 %	33⋅2 %
Woke up feeling unrested (≥3 nights)	66⋅3 %	58⋅4 %	51⋅2 %	40⋅5 %	43⋅0 %
Sleep medication (≥3 nights)	21⋅9 %	14⋅6 %	12⋅2 %	8⋅8 %	9⋅8 %
**Clinical characteristics**	**Very Low**	**Low**	**Marginal**	**High**	**Overall**
Health status					
Excellent/very good	24⋅0 %	31⋅1 %	39⋅0 %	62⋅9 %	58⋅4 %
Good	29⋅4 %	31⋅7 %	33⋅9 %	26⋅4 %	27⋅5 %
Fair/poor	46⋅6 %	37⋅1 %	27⋅1 %	10⋅6 %	14⋅2 %
Severe psychological distress^d^	21⋅5 %	10⋅5 %	7⋅3 %	2⋅0 %	3⋅5 %
Body mass index (BMI)					
Recommended (18⋅5 to <25 km/m^2^)	28⋅5 %	26⋅2 %	27⋅2 %	34⋅0 %	33⋅0 %
Overweight (25–29⋅9 km/m^2^)	29⋅4 %	32⋅2 %	33⋅8 %	36⋅6 %	35⋅9 %
Obese (≥30 kg/m^2^)	42⋅1 %	41⋅5 %	39⋅0 %	29⋅4 %	31⋅1 %
Dyslipidaemia^e^	58⋅5 %	53⋅9 %	49⋅8 %	49⋅0 %	49⋅7 %
Hypertension^f^	50⋅3 %	45⋅9 %	41⋅2 %	34⋅1 %	35⋅7 %
Prediabetes^g^	8⋅3 %	7⋅5 %	7⋅3 %	6⋅3 %	6⋅5 %
Type 2 Diabetes Mellitus^h^	21⋅7 %	20⋅4 %	16⋅5 %	9⋅9 %	11⋅2 %
‘Ideal’ cardiovascular health^i^	3⋅6 %	5⋅2 %	6⋅4 %	12⋅6 %	11⋅4 %

se, standard error.

a Notes: All estimates are weighted for the survey's complex sampling design. All estimates are age-standardised to the US 2010 population, except for age. Percentage may not sum to 100 due to missing values or rounding.

b Meets PA guidelines defined as ≥150 min/week of moderate intensity or ≥75 min/week of vigorous intensity or ≥150 min/week of moderate and vigorous intensity.

c Insomnia symptoms defined as either trouble falling or staying asleep 3+ days a week.

d Kessler 6-psychological distress scale score ≥13.

e Dyslipidaemia defined as high cholesterol in the 12 months prior to interview. Available for survey years 2011–2017.

f Hypertension defined as ever told by a doctor had hypertension.

g Prediabetes defined as ever told by a doctor had prediabetic condition.

h Type 2 Diabetes Mellitus defined as ever told by a doctor or health professional that you have diabetes or sugar diabetes.

i ‘Ideal’ cardiovascular health includes never smoking/quit >12 months prior to interview, BMI 18⋅5 to <25 kg/m^2^, meeting physical activity guidelines, and no prior diagnosis of dyslipidemia, hypertension, or prediabetes.

Most participants (64⋅4 %) reported the recommended hours of sleep (7–9 h). The prevalence of recommended sleep was greatest in households with high food security status (66⋅7 %) compared with marginal (55⋅9 %), low (53⋅1 %) and very low (42⋅7 %). The most reported sleep problem was waking up feeling unrested (43⋅0 %) followed by insomnia symptoms (33⋅2 %), trouble staying asleep (27⋅4 %), trouble falling asleep (20⋅1 %) and taking sleep medications ≥3 nights per week (9⋅8 %). The prevalence of sleep disturbances was highest among those living in households with very low food security status and subsequently decreased as food security status increased. For example, those living in households with very low and low food security status had a higher percentage of very short sleep (23⋅6 and 15⋅5 %, respectively) compared to those with high food security status (7⋅7 %) (Table 1).

**Fig. 1.**
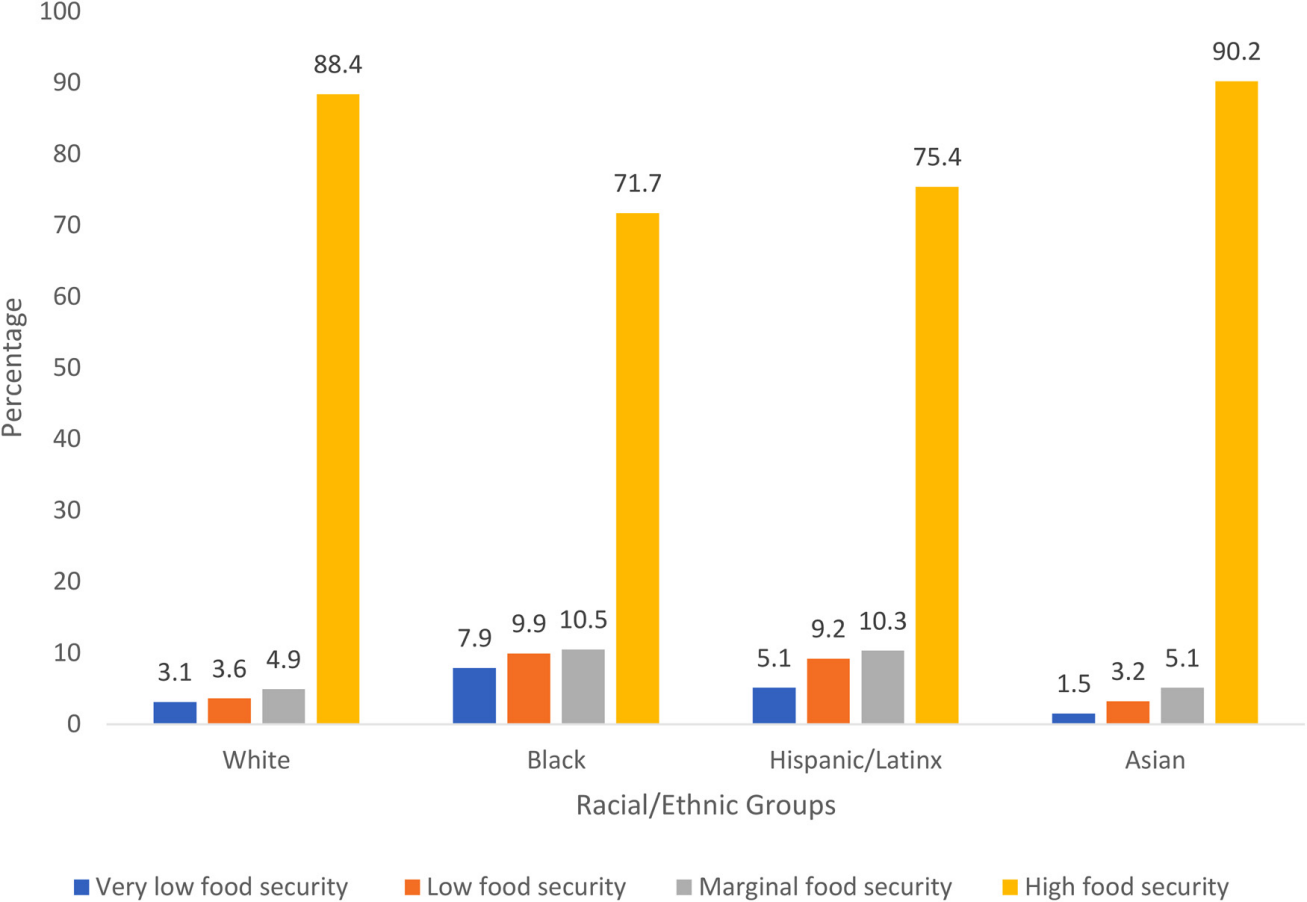
Food security status by race/ethnicity between very low, low, marginal and high food security, National Health Interview Survey, 2013–2018 (*N* 177 435).

A higher percent of NH-Black (7⋅9 %) and Hispanic/Latinx (5⋅1 %) lived in households with very low food security compared with NH-White (3⋅1 %) participants (Fig. 1). Likewise, a higher percent of NH-Black (9⋅9 %) and Hispanic/Latinx (9⋅2 %) lived in households with low food security compared with NH-White (3⋅6 %) and NH-Asian (3⋅2 %) (Fig. 1).

NH-Black (24⋅3 %) and Hispanic/Latinx (20⋅5 %) participants reported higher percentage of being worried whether food would run out before they got money to buy more compared with NH-White (9⋅5 %) participants (Supplementary Table S1). Women reported a higher percentage living in households with very low food security (59⋅1 %) compared with 40⋅9 % of men (Supplementary Figure S2).

Participants living in households with very low and low food security status had a higher percentage of very short sleep (9⋅7 and 8⋅6 %, respectively), short sleep (6⋅0 and 6⋅6 %, respectively) and long sleep (7⋅4 and 8⋅4 %, respectively) compared with recommended sleep (2⋅5 and 4⋅2 %, respectively) (Supplementary Figure S3).

### Food insecurity and sleep health

Overall, participants living in households with very low food security *v.* high food security had a higher prevalence of very short sleep (PR = 2⋅61 [95 % CI 2⋅44–2⋅80]), trouble falling asleep (PR = 2⋅21 [95 % CI 2⋅12–2⋅30]) and using sleep medication (PR = 2⋅22 [95 % CI 2⋅07–2⋅37]), after adjustment (Table 2). Similar patterns emerged with low and marginal *v.* high food security where point estimates were higher in low *v.* high compared with marginal *v.* high food security (Table 2).

**Table 2.** Prevalence ratios of sleep health among participants reporting very low, low and marginal compared with high food security by sex/gender and race/ethnicity, National Health Interview Survey, 2013–2018 (*N* 177 435)

Sleep health dimensions								
Food security	<6 *v*. 7–9 h	<7 *v*. 7–9 h	>9 *v*. 7–9 h	Trouble falling asleep (≥3 nights)	Trouble staying asleep (≥3 nights)	Insomnia symptoms^a^	Woke up feeling unrested (≥3 days)	Sleep medication (≥3 nights)
Overall *n* 177 435								
Very low	**2⋅61 (2⋅44–2⋅80)**	**1⋅66 (1⋅60–1⋅72)**	**1⋅54 (1⋅35–1⋅75)**	**2⋅21 (2⋅12–2⋅30)**	**1⋅98 (1⋅91–2⋅06)**	**1⋅88 (1⋅82–1⋅94)**	**1⋅62 (1⋅58–1⋅66)**	**2⋅22 (2⋅07–2⋅37)**
Low	**1⋅74 (1⋅62–1⋅86)**	**1⋅33 (1⋅29–1⋅38)**	**1⋅26 (1⋅11–1⋅43)**	**1⋅71 (1⋅63–1⋅78)**	**1⋅55 (1⋅49–1⋅61)**	**1⋅52 (1⋅47–1⋅57)**	**1⋅44 (1⋅40–1⋅48)**	**1⋅59 (1⋅47–1⋅71)**
Marginal	**1⋅51 (1⋅41–1⋅63)**	**1⋅24 (1⋅20–1⋅29)**	**1⋅25 (1⋅10–1⋅42)**	**1⋅46 (1⋅39–1⋅52)**	**1⋅37 (1⋅32–1⋅43)**	**1⋅37 (1⋅33–1⋅42)**	**1⋅27 (1⋅24–1⋅30)**	**1⋅37 (1⋅27–1⋅47)**
Women *n* 97 493 (55 %)								
Very low	**2⋅61 (2⋅41–2⋅82)**	**1⋅66 (1⋅59–1⋅74)**	**1⋅52 (1⋅28–1⋅79)**	**2⋅14 (2⋅04–2⋅25)**	**1⋅90 (1⋅82–1⋅99)**	**1⋅80 (1⋅74–1⋅87)**	**1⋅60 (1⋅56–1⋅65)**	**2⋅25 (2⋅07–2⋅44)**
Low	**1⋅80 (1⋅66–1⋅96)**	**1⋅32 (1⋅26–1⋅38)**	**1⋅23 (1⋅04–1⋅45)**	**1⋅66 (1⋅58–1⋅75)**	**1⋅54 (1⋅47–1⋅61)**	**1⋅49 (1⋅43–1⋅55)**	**1⋅44 (1⋅40–1⋅48)**	**1⋅57 (1⋅43–1⋅72)**
Marginal	**1⋅52 (1⋅38–1⋅66)**	**1⋅25 (1⋅19–1⋅30)**	1⋅15 (0⋅96–1⋅37)	**1⋅45 (1⋅37–1⋅53)**	**1⋅38 (1⋅31–1⋅45)**	**1⋅36 (1⋅31–1⋅42)**	**1⋅27 (1⋅24–1⋅31)**	**1⋅45 (1⋅33–1⋅59)**
Men *n* 79 942 (45 %)								
Very low	**2⋅62 (2⋅36–2⋅91)**	**1⋅65 (1⋅56–1⋅74)**	**1⋅55 (1⋅27–1⋅91)**	**2⋅32 (2⋅16–2⋅49)**	**2⋅11 (1⋅98–2⋅25)**	**2⋅00 (1⋅89–2⋅11)**	**1⋅64 (1⋅57–1⋅71)**	**2⋅16 (1⋅91–2⋅43)**
Low	**1⋅66 (1⋅47–1⋅86)**	**1⋅35 (1⋅28–1⋅43)**	**1⋅27 (1⋅04–1⋅54)**	**1⋅78 (1⋅64–1⋅92)**	**1⋅54 (1⋅45–1⋅46)**	**1⋅55 (1⋅47–1⋅65)**	**1⋅43 (1⋅37–1⋅50)**	**1⋅60 (1⋅40–1⋅84)**
Marginal	**1⋅50 (1⋅34–1⋅68)**	**1⋅24 (1⋅17–1⋅31)**	**1⋅38 (1⋅14–1⋅67)**	**1⋅47 (1⋅37–1⋅59)**	**1⋅36 (1⋅27–1⋅46)**	**1⋅39 (1⋅32–1⋅46)**	**1⋅27 (1⋅21–1⋅32)**	**1⋅20 (1⋅04–1⋅40)**
NH-White *n* 118 609 (66⋅9 %)								
Very low	**2⋅73 (2⋅50–2⋅99)**	**1⋅71 (1⋅63–1⋅80)**	**1⋅74 (1⋅47–2⋅05)**	**2⋅11 (2⋅00–2⋅23)**	**1⋅94 (1⋅85–2⋅02)**	**1⋅81 (1⋅74–1⋅88)**	**1⋅63 (1⋅58–1⋅68)**	**2⋅04 (1⋅87–2⋅21)**
Low	**1⋅97 (1⋅80–2⋅16)**	**1⋅44 (1⋅37–1⋅50)**	**1⋅28 (1⋅07–1⋅52)**	**1⋅72 (1⋅63–1⋅82)**	**1⋅58 (1⋅50–1⋅65)**	**1⋅51 (1⋅46–1⋅58)**	**1⋅49 (1⋅45–1⋅54)**	**1⋅56 (1⋅42–1⋅71)**
Marginal	**1⋅72 (1⋅56–1⋅89)**	**1⋅32 (1⋅27–1⋅39)**	**1⋅19 (1⋅01–1⋅41)**	**1⋅47 (1⋅39–1⋅55)**	**1⋅39 (1⋅33–1⋅45)**	**1⋅37 (1⋅32–1⋅42)**	**1⋅30 (1⋅26–1⋅35)**	**1⋅38 (1⋅26–1⋅51)**
NH-Black *n* 22 356 (12⋅6 %)								
Very low	**2⋅03 (1⋅80–2⋅31)**	**1⋅44 (1⋅34–1⋅55)**	1⋅19 (0⋅94–1⋅52)	**2⋅03 (1⋅84–2⋅23)**	**1⋅78 (1⋅63–1⋅94)**	**1⋅75 (1⋅63–1⋅89)**	**1⋅54 (1⋅45–1⋅64)**	**2⋅31 (1⋅96–2⋅73)**
Low	**1⋅44 (1⋅26–1⋅64)**	**1⋅23 (1⋅14–1⋅32)**	**1⋅37 (1⋅08–1⋅74)**	**1⋅56 (1⋅41–1⋅73)**	**1⋅45 (1⋅33–1⋅59)**	**1⋅42 (1⋅31–1⋅53)**	**1⋅40 (1⋅31–1⋅49)**	**1⋅65 (1⋅39–1⋅97)**
Marginal	**1⋅19 (1⋅03–1⋅38)**	**1⋅12 (1⋅04–1⋅22)**	1⋅26 (0⋅95–1⋅67)	**1⋅28 (1⋅15–1⋅44)**	**1⋅24 (1⋅11–1⋅38)**	**1⋅24 (1⋅13–1⋅36)**	**1⋅21 (1⋅13–1⋅30)**	**1⋅27 (1⋅02–1⋅58)**
Hispanic/Latinx *n* 26 709 (15⋅1 %)								
Very low	**2⋅65 (2⋅30–3⋅07)**	**1⋅65 (1⋅53–1⋅79)**	**1⋅63 (1⋅19–2⋅22)**	**2⋅47 (2⋅24–2⋅73)**	**2⋅18 (1⋅97–2⋅41)**	**2⋅12 (1⋅95–2⋅31)**	**1⋅57 (1⋅48–1⋅67)**	**2⋅76 (2⋅32–3⋅29)**
Low	**1⋅54 (1⋅32–1⋅81)**	**1⋅20 (1⋅10–1⋅31)**	1⋅22 (0⋅90–1⋅67)	**1⋅73 (1⋅57–1⋅91)**	**1⋅45 (1⋅31–1⋅60)**	**1⋅58 (1⋅45–1⋅71)**	**1⋅34 (1⋅27–1⋅42)**	**1⋅67 (1⋅33–2⋅08)**
Marginal	**1⋅24 (1⋅06–1⋅46)**	**1⋅13 (1⋅04–1⋅22)**	**1⋅41 (1⋅03–1⋅92)**	**1⋅51 (1⋅36–1⋅67)**	**1⋅39 (1⋅25–1⋅55)**	**1⋅47 (1⋅34–1⋅61)**	**1⋅21 (1⋅14–1⋅29)**	**1⋅33 (1⋅07–1⋅65)**
NH-Asian *n* 9761 (5⋅5 %)								
Very low	**3⋅64 (2⋅67–4⋅97)**	**1⋅89 (1⋅59–2⋅24)**	1⋅01 (0⋅22–4⋅69)	**2⋅74 (2⋅04–3⋅68)**	**2⋅69 (2⋅05–3⋅55)**	**2⋅16 (1⋅68–2⋅77)**	**1⋅85 (1⋅57–2⋅18)**	**2⋅68 (1⋅49–4⋅79)**
Low	**2⋅04 (1⋅38–3⋅02)**	**1⋅56 (1⋅30–1⋅89)**	0⋅52 (0⋅18–1⋅47)	**2⋅01 (1⋅49–2⋅71)**	**2⋅45 (1⋅91–3⋅13)**	**1⋅96 (1⋅57–2⋅46)**	**1⋅51 (1⋅27–1⋅79)**	0⋅74 (0⋅33–1⋅65)
Marginal	**1⋅80 (1⋅29–2⋅52)**	**1⋅33 (1⋅14–1⋅55)**	1⋅17 (0⋅63–2⋅19)	**1⋅57 (1⋅20–2⋅05)**	**1⋅56 (1⋅22–2⋅00)**	**1⋅59 (1⋅30–1⋅94)**	**1⋅28 (1⋅11–1⋅48)**	1⋅67 (0⋅94–2⋅94)

Overall model adjusted for age (18–30, 31–50, ≥50), sex/gender (women or men), race/ethnicity (NH-White, NH-Black, Hispanic/Latinx and NH-Asian/PI), educational attainment (<high school, high school graduate, some college, ≥college), annual household income (<$35 000, $35 000–$74 999, $75 000+), occupational class (professional/management, support services, labourers), region of residence (Northeast, Midwest, South, West), marital/co-habiting status(married/living with partner or co-habitating, divorced/widowed/separated, single/no live-in partner) and employment status (unemployed, employed).

Reference level: high food security.

Note. All estimates are weighted for the survey's complex sampling design. Boldface indicates statistically significant results at the 0⋅05 level.

a Insomnia symptoms defined as either trouble staying or falling asleep 3+ times a week.

Interaction term between food security status and race/ethnicity was statistically significant (*P*-value < 0⋅00001) but was not between food security status and sex/gender (*P*-value = 0⋅1059).

### Food insecurity and sleep health by sex/gender

Men living in households with very low *v.* high food insecurity had a higher prevalence of trouble staying asleep (PR = 2⋅11 [95 % CI 1⋅98–2⋅25]) and insomnia symptoms (PR = 2⋅00 [95 % CI 1⋅89–2⋅11]) than women (PR = 1⋅90 [95 % CI 1⋅82–1⋅99]; PR = 1⋅80 [95 % CI 1⋅74–1⋅87], respectively), after adjustment (Table 2). Both men and women living in households with very low, low and marginal *v.* high food security status had a similar prevalence of sleep duration, trouble falling asleep, waking up feeling unrested and using sleep medications. For example, very low *v.* high food security was associated with an over two-fold time the prevalence of very short sleep among men (PR = 2⋅62 [95 % CI 2⋅36–2⋅91]) and women (PR = 2⋅61 [95 % CI 2⋅41–2⋅82]) (Table 2).

### Food insecurity and sleep health by race/ethnicity

Living in households with very low *v.* high food security status was associated with very short sleep duration among NH-Asian (PR = 3⋅64 [95 % CI 2⋅67–4⋅97]), NH-White (PR = 2⋅73 [95 % CI 2⋅50–2⋅99]), Hispanic/Latinx (PR = 2⋅65 [95 % CI 2⋅30–3⋅07]) and NH-Black (PR = 2⋅03 [95 % CI 1⋅80–2⋅31]) adults, after adjustment (Table 2). Similar patterns emerged with very low *v.* high food security status and short sleep duration across racial/ethnic groups.

Living in households with low *v.* high food security status was associated with very short sleep duration among NH-Asian (PR = 2⋅04 [95 % CI 1⋅38–3⋅02]), NH-White (PR = 1⋅97 [95 % CI 1⋅80–2⋅16]), Hispanic/Latinx (PR = 1⋅54 [95 % CI 1⋅32–1⋅81]) and NH-Black (PR = 1⋅44 [95 % CI 1⋅26–1⋅64]), after adjustment. Similar patterns emerged with low *v.* high food security status and short sleep duration across racial/ethnic groups, although the point estimate was higher in NH-Black than Hispanic/Latinx participants. There was no statistically significant modification by race/ethnicity for low *v.* high food security and long sleep duration.

Living in households with marginal food security status was associated with very short sleep duration among NH-Asian (PR = 1⋅80 [95 % CI 1⋅29–2⋅52]), NH-White (PR = 1⋅72 [95 % CI 1⋅56–1⋅89]), Hispanic/Latinx (PR = 1⋅24 [95 % CI 1⋅06–1⋅46]) and NH-Black (PR = 1⋅19 [95 % CI 1⋅03–1⋅38]) counterparts with high food security, after adjustment. Similar patterns emerged with marginal *v.* high food security status and short sleep duration across racial/ethnic groups. There was no interaction by race/ethnicity for marginal *v.* high food security and long sleep duration.

Hispanic/Latinx participants who lived in households with very low *v.* high food security status had 2⋅47 (95 % CI 2⋅24–2⋅73) times the prevalence of trouble falling asleep, 2⋅18 (95 % CI 1⋅97–2⋅31) times the prevalence of trouble staying asleep, 2⋅12 (95 % CI 1⋅95–2⋅31) times the prevalence of insomnia symptoms and 2⋅76 (95 % CI 2⋅32–3⋅29) times the prevalence of using of sleep medications, after adjustment. NH-Asian participants who lived in households with very low *v.* high food security status had 2⋅69 (95 % CI 2⋅05–3⋅55) times the prevalence of trouble staying asleep, after adjustment (Table 2).

### Food insecurity and sleep health by minoritised racial/ethnic groups compared with NH-White participants with high food security

Compared with NH-White participants with high food security, NH-Black participants who lived in households with very low (PR = 2⋅81 [95 % CI 2⋅50–3⋅16]), low (PR = 1⋅94 [95 % CI 1⋅72–2⋅19]) and marginal (PR = 1⋅66 [95 % CI 1⋅44–1⋅92]) food security had a higher prevalence of very short sleep duration (Table 3). Among Hispanic/Latinx participants, living in households with very low food security was associated with a higher prevalence of very short (PR = 2⋅22 [95 % CI 1⋅92–2⋅56]) and short sleep duration (PR = 1⋅50 [95 % CI 1⋅39–1⋅61]) as well as more sleep disturbances. For example, Hispanic/Latinx living in households with very low food security had an 83 % (PR = 1⋅83 [95 % CI 1⋅67–2⋅01]) higher prevalence of trouble falling asleep compared with NH-Whites with high food security. Among NH-Asian participants, living in households with very low (PR = 3⋅75 [95 % CI 2⋅83–4⋅95]), low (PR = 2⋅07 [95 % CI 1⋅41–3⋅02]), marginal (PR = 1⋅81 [95 % CI 1⋅32–2⋅47]) and high (PR = 1⋅18 [95 % CI 1⋅05–1⋅32]) food security was associated with a higher prevalence of very short sleep duration.

**Table 3.** Prevalence ratios of sleep health among minoritised racial/ethnic groups reporting very low, low, marginal and high compared with NH-White participants with high food security, National Health Interview Survey, 2013–2018 (*N* 177 435)

Sleep health dimensions								
Food security	<6 *v*. 7–9 h	<7 *v*. 7–9 h	>9 *v*. 7–9 h	Trouble falling asleep (≥3 nights)	Trouble staying asleep (≥3 nights)	Insomnia symptoms^a^	Woke up feeling unrested (≥3 days)	Sleep medication (≥3 nights)
NH-Black *n* 22 356 (12⋅6 %)								
Very low	**2⋅81 (2⋅50–3⋅16)**	**1⋅74 (1⋅63–1⋅86)**	1⋅26 (1⋅00–1⋅59)	**1⋅65 (1⋅52–1⋅79)**	**1⋅49 (1⋅39–1⋅60)**	**1⋅43 (1⋅34–1⋅53)**	**1⋅38 (1⋅31–1⋅46)**	**1⋅25 (1⋅08–1⋅45)**
Low	**1⋅94 (1⋅71–2⋅19)**	**1⋅47 (1⋅38–1⋅58)**	**1⋅53 (1⋅21–1⋅93)**	**1⋅25 (1⋅14–1⋅37)**	**1⋅19 (1⋅09–1⋅29)**	**1⋅14 (1⋅06–1⋅22)**	**1⋅25 (1⋅18–1⋅32)**	0⋅88 (0⋅76–1⋅03)
Marginal	**1⋅66 (1⋅44–1⋅92)**	**1⋅38 (1⋅28–1⋅48)**	**1⋅48 (1⋅13–1⋅95)**	1⋅03 (0⋅92–1⋅14)	1⋅01 (0⋅91–1⋅12)	0⋅99 (0⋅91–1⋅08)	**1⋅09 (1⋅02–1⋅16)**	**0⋅67 (0⋅55–0⋅82)**
High	**1⋅72 (1⋅59–1⋅86)**	**1⋅34 (1⋅30–1⋅39)**	**1⋅30 (1⋅16–1⋅47)**	**0⋅84 (0⋅79–0⋅89)**	**0⋅81 (0⋅76–0⋅85)**	**0⋅79 (0⋅76–0⋅83)**	**0⋅96 (0⋅93–0⋅99)**	**0⋅51 (0⋅46–0⋅57)**
Hispanic/Latinx *n* 26 709 (15⋅1 %)								
Very low	**2⋅22 (1⋅92–2⋅56)**	**1⋅50 (1⋅39–1⋅61)**	1⋅24 (0⋅92–1⋅67)	**1⋅83 (1⋅67–2⋅01)**	**1⋅49 (1⋅36–1⋅63)**	**1⋅47 (1⋅36–1⋅59)**	**1⋅37 (1⋅29–1⋅45)**	**1⋅50 (1⋅29–1⋅75)**
Low	1⋅17 (0⋅99–1⋅38)	1⋅06 (0⋅97–1⋅16)	0⋅89 (0⋅66–1⋅19)	**1⋅23 (1⋅12–1⋅36)**	0⋅94 (0⋅85–1⋅04)	1⋅05 (0⋅96–1⋅14)	**1⋅16 (1⋅09–1⋅23)**	0⋅82 (0⋅67–1⋅01)
Marginal	0⋅98 (0⋅83–1⋅16)	1⋅02 (0⋅94–1⋅10)	1⋅07 (0⋅80–1⋅45)	1⋅09 (0⋅99–1⋅20)	0⋅90 (0⋅81–1⋅00)	0⋅98 (0⋅91–1⋅07)	1⋅05 (0⋅99–1⋅11)	**0⋅65 (0⋅53–0⋅79)**
High	0⋅98 (0⋅90–1⋅08)	1⋅02 (0⋅98–1⋅06)	0⋅82 (0⋅71–0⋅95)	**0⋅80 (0⋅76–0⋅85)**	**0⋅69 (0⋅66–0⋅73)**	**0⋅72 (0⋅69–0⋅75)**	**0⋅93 (0⋅90–0⋅96)**	**0⋅53 (0⋅48–0⋅59)**
NH-Asian *n* 9761 (5⋅5 %)								
Very low	**3⋅75 (2⋅83–4⋅95)**	**2⋅05 (1⋅75–2⋅40)**	1⋅13 (0⋅24–5⋅35)	**1⋅77 (1⋅36–2⋅32)**	**1⋅48 (1⋅16–1⋅89)**	**1⋅32 (1⋅06–1⋅65)**	**1⋅51 (1⋅30–1⋅75)**	**1⋅34 (0⋅80–2⋅24)**
Low	**2⋅07 (1⋅41–3⋅02)**	**1⋅64 (1⋅37–1⋅95)**	0⋅61 (0⋅24–1⋅56)	1⋅29 (0⋅98–1⋅69)	**1⋅27 (1⋅00–1⋅61)**	1⋅13 (0⋅91–1⋅40)	**1⋅22 (1⋅03–1⋅44)**	**0⋅31 (0⋅14–0⋅68)**
Marginal	**1⋅81 (1⋅32–2⋅47)**	**1⋅41 (1⋅22–1⋅63)**	1⋅14 (0⋅63–2⋅06)	0⋅92 (0⋅72–1⋅18)	**0⋅78 (0⋅61–0⋅99)**	0⋅87 (0⋅72–1⋅05)	1⋅04 (0⋅91–1⋅19)	0⋅60 (0⋅35–1⋅01)
High	**1⋅18 (1⋅05–1⋅32)**	**1⋅12 (1⋅06–1⋅18)**	0⋅91 (0⋅72–1⋅13)	**0⋅61 (0⋅56–0⋅67)**	**0⋅50 (0⋅47–0⋅54)**	**0⋅55 (0⋅52–0⋅59)**	**0⋅85 (0⋅82–0⋅89)**	**0⋅33 (0⋅27–0⋅40)**

Model adjusted for age (18–30, 31–50, ≥50), sex/gender (women or men), educational attainment (<high school, high school graduate, some college, ≥college), annual household income (<$35 000, $35 000–$74 999, $75 000+), occupational class (professional/management, support services, labourers), region of residence (Northeast, Midwest, South, West), marital/co-habiting status(married/living with partner or co-habitating, divorced/widowed/separated, single/no live-in partner) and employment status (unemployed, employed).

Reference level: NH-White with high food security.

Note. All estimates are weighted for the survey's complex sampling design. Boldface indicates statistically significant results at the 0⋅05 level.

a Insomnia symptoms defined as either trouble staying or falling asleep 3+ times a week.

Compared with NH-White participants living in households with high food security, minoritised racial/ethnic groups had lower sleep disturbances. For example, NH-Black (PR = 0⋅79 [95 % CI 0⋅76–0⋅83]), Hispanic/Latinx (PR = 0⋅72 [95 % CI 0⋅69–0⋅75]) and NH-Asian (PR = 0⋅55 [95 % CI 0⋅52–0⋅59]) participants living in households with high food security status had less insomnia symptoms.

## Discussion

In our large nationally representative, racially/ethnically diverse sample of the US population, the prevalence of food insecurity was highest among NH-Black adults followed by Hispanic/Latinx, NH-White and NH-Asian adults. We also found that living in households with lower food security status was associated with poorer sleep health, which aligned with our hypothesis. While we observed a similar prevalence of sleep duration among women and men living in households with very low *v.* high food security status, there was a higher prevalence of sleep disturbances among men compared with women contrary to our hypothesis. Also inconsistent with our hypothesis, we reported stronger associations between food insecurity and very short as well as short sleep duration among NH-Asian and NH-White adults than Hispanic/Latinx and NH-Black adults. However, stronger associations were observed among Hispanic/Latinx living in households with very low *v.* high food security status and sleep disturbances (e.g. trouble falling asleep). Furthermore, we found a ‘dose response’ relationship between very low, low and marginal food security status and sleep duration among minoritised racially/ethnically adults (e.g. NH-Black adults) *v*. NH-White adults living in households with high food security status, consistent with our hypothesis.

Similar to prior literature, we observed participants living in households with lower *v.* higher food security status was associated with poorer sleep health^(17)^ including in studies of college students^(20)^ and adolescents^(25)^ as well as studies using objectively measured sleep dimensions^(16)^. For example, a study using data from BRFSS (Behavioral Risk Factor Surveillance System) across twelve states found that the prevalence of insufficient sleep was significantly higher among food insecure individuals^(21)^. A meta-analysis of eight cross-sectional studies reported food insecurity *v.* security was associated with increased odds of sleep disorders, such as insomnia symptoms^(34)^. Another recent meta-analysis found severity of food insecurity levels to be associated with poorer sleep quality, including trouble falling asleep, trouble staying asleep and shorter sleep duration^(35)^.

Even though few studies have examined the food security status–sleep health relationship by sex/gender, our findings corroborate the currently scant prior literature^(17)^. For example, a NHANES (National Health and Nutrition Examination Survey) study found women but not men with very low *v.* high food security reported significantly shorter sleep duration^(17)^. Our NHIS study expands on these results by demonstrating this association among individuals with very low *v.* high food security and higher prevalence of both very short and short sleep duration. This demonstrates that individuals on the margins of society (i.e. very low food security; very short sleep) are most impacted by disadvantage. Moreover, our findings further expand on these results by illustrating both women and men, and not only women^(17)^, with very low *v.* high food security status had a higher prevalence of shorter sleep duration. Because of our larger sample size (177 435 *v*. 10 901), our study was more likely able to detect meaningful differences among men. Therefore, the threshold appeared different for men, which is important to consider when designing interventions.

While there were stronger associations of sleep duration (e.g. very short sleep) among NH-White and NH-Asian adults living in households with very low *v.* high food security status compared with Hispanic/Latinx and NH-Black adults, we observed both higher levels of food insecurity and poor sleep health measures among NH-Black and Hispanic/Latinx adults. Of note, we reported NH-Asian adults with very low *v.* high food security status had the highest prevalence of very short sleep duration compared with other racial/ethnic groups. This finding fills a gap in the literature by demonstrating an association between food insecurity *v.* security and poorer sleep among NH-Asians. Nevertheless, due to the diversity of this group (e.g. South Asian; East Asian), future studies should be replicated among subgroup Asian populations to identify groups most impacted. Another interesting finding and in alignment with our hypothesis, Hispanic/Latinx living in households with very low *v.* high food security status had a higher prevalence of sleep disturbances including trouble falling asleep, trouble staying asleep and insomnia symptoms. Our results are consistent with the few studies that have examined the food security status and sleep health relationship by racial/ethnic groups in a large population^(24)^. For example, a study among 2172 adults with obesity and high levels of food insecurity had more trouble falling asleep among minoritised racial/ethnic groups including NH-Black, Hispanic and NH-Asian as well as other racial/ethnic groups^(22)^. Given the documentation of both food insecurity and poor sleep health disproportionately impacting minoritised racial/ethnic groups^(18,36)^, these results are unsurprising. Similarly, another study among first-generation US College students comprised of approximately 40 % of minoritised racial/ethnic groups found those food insecure *v.* secure had higher odds of poorer sleep quality measured via the Pittsburgh Sleep Quality Index^(37)^. A recent study among American Indian/Alaskan Native youth also found that higher food insecurity was associated with more sleep disturbances^(38)^.

Food insecurity may influence sleep through several proposed mechanisms, including biological, psychological and social. Adults who are food insecure *v.* secure are less likely to access and consume fruits, vegetables and protein and are more likely to consume sugar-sweetened beverages. Lower diet quality, inadequate nutrient intake and poor nutrition likely contributes to shorter sleep duration^(7)^. Being food insecure may also lead to poor sleep through restricted caloric intake where hunger may interfere with sleep quality^(39)^. Worrying about when one's next meal is and/or ability to afford one's next meal as well as other psychological distress may negatively impact sleep^(9,34)^. During public health crises, such as the ongoing COVID-19 pandemic, psychological distress is heightened and disproportionately impacts minoritised racial/ethnic groups^(40)^, which may further impact sleep. In fact, a recent study found that psychological distress was exacerbated during the pandemic where people with food insecurity *v*. security had higher anxiety and depression^(41)^. Moreover, a recent meta-analysis comprised of 250 studies from 49 countries estimated higher levels of sleep disturbances during the COVID-19 pandemic compared to before, disproportionately impacting those infected with COVID-19, older adults, children and healthcare workers^(42)^. Another meta-analysis reported similar levels of heighted sleep disturbances as well as circadian disruptions during other infectious disease outbreaks (e.g. Influenza, Ebola and Zika)^(43)^. Heightened levels of stress activate the HPA axis producing a range of neuroendocrine hormones, such as corticotropin-releasing hormone, and thus may impact sleep^(8)^. The mental and emotional toll of living in poverty may also activate the HPA axis^(44)^. Other societal mechanisms that may impact sleep include food deserts or swamps where minoritised racial/ethnic groups are more likely to live in areas with limited to no access to fresh and healthy foods^(18,45)^ and environmental pollution, such as noise, that impact optimal sleep^(46)^. Climate change may also impact sleep through food insecurity where climate change has been shown to disrupt agricultural systems and food supply by, for example, rising temperatures resulting in crop failure and constraining supply^(47)^.

There were several limitations to our study including the cross-sectional study design that limits ability to infer causality. We also relied on self-reported data with known measurement error, including sleep, which has been shown to be non-differential across racial/ethnic groups^(48,49)^. Future studies should also include objective measures. The food security status questions were based on a respondent answering on behalf of the household, and therefore, we were unable to understand intra-household dynamics in terms of who is affected by food insecurity. There is also potential for residual confounding since we adjusted for some measures relatively crudely, such as employment status *v*. a more refined occupational measure that is not available in the NHIS. Additionally, the response rate is relatively low although it is comparable or higher than other national surveillance systems used to monitor the health of the US population. Furthermore, we were unable to account for transgender and non-binary individuals as the NHIS uses a binary definition of sex/gender. Future research should examine the intersection of multiple social categories including race/ethnicity, sex/gender, age and annual household income. Moreover, future research should replicate this study among indigenous populations as they are considered among the most vulnerable, yet least studied populations facing food insecurity^(50)^. Longitudinal studies with participants of all age ranges can enhance our understanding of the prospective impact of food insecurity across the life course as well as help elucidate causal mechanisms.

Despite these limitations, our study had important strengths including utilising a nationally representative dataset with a large sample size. Our results are generalisable to the NH-White, NH-Black, Hispanic/Latinx and NH-Asian US populations. Furthermore, our racially/ethnically diverse sample allowed us to examine the relationship between food security status and sleep health among NH-Asians, which is limited in current research as most studies do not consider racial/ethnic differences in food security status and sleep health^(17,20,21)^ and even fewer include NH-Asians^(22–24)^. Another strength includes using multiple dimensions of sleep health beyond sleep duration (e.g. insomnia symptoms, waking up feeling unrested) as well as additional parameters within sleep duration (e.g. very short, short). Additionally, we used a recommended scale^(28)^, where short survey forms of HFSSM have been validated^(51)^, to assess multiple domains of food security status, such as food access, food intake and food affordability, whereas most studies only ask one^(25)^ or three questions^(22)^.

Given study findings identified NH-Black, Hispanic/Latinx and NH-Asian adults most impacted by food insecurity, these results illuminate who needs most access to high-quality food and resources to reduce food insecurity–sleep disparities. Our descriptive findings inform the need for resource allocation to minoritised racial/ethnic groups along with policy, programme and research development, strengthening and/or enforcement^(52)^. While we did not sample children, it has been well-documented that food assistance programmes (e.g. Supplemental Nutrition Assistance Program (SNAP); National School Breakfast and Lunch Programs) are vital for low-income children and can also simultaneously reduce food insecurity among their parents. During the COVID-19 pandemic, the necessity of food nutrition programmes in schools and communities (e.g. SNAP) were highlighted^(53,54)^. Previous research has documented that food insecurity is higher in the summer months, particularly among racial/ethnic minoritised children^(54)^. One study in Philadelphia, Pennsylvania projected that – during short-term emergencies that impact household food access (e.g. disaster, hurricane, pandemic) – 3 d of school closures could result in more than 400 000 missed meals for children^(55)^. As public health crises are likely to worsen due to issues related to climate change for instance, it is essential to seek other effective alternative strategies including the expansion of federal assistance programmes (including eligibility), local grocery stores, all year meal programmes and innovations (e.g. open to community members; food bank/pantry partnerships), community gardens and improving public transportation. Since previous studies have reported individuals living in rural and poor areas have limited access to food due to transportation^(56)^, legislation can be used to improve transportation to help close the gap in food security. For example, an affordable and publicly run Grocery Bus line that was assimilated into the regular transit system in Austin, Texas intentionally connected a low-income Latinx community with insufficient transportation limiting supermarket access^(56)^. Finally, policies mitigating climate change may also help improve food security status^(47)^. With this approach, we can build towards not only food security but also nutritional security where all people have access to sufficient, nutritious and high-quality food at all times^(1)^.

Ultimately, we found that the prevalence of low food security was high among a racially/ethnically diverse sample of the US population with the highest prevalence among NH-Black adults. We also found that low *v.* high food security status was associated with multiple dimensions of poor sleep health. Rising economic and public health crises, as influenced by the COVID-19 pandemic and climate change, demonstrate the urgent need to address food insecurity among minoritised racial/ethnic groups as existing racial/ethnic disparities will likely persist and worsen^(53)^. Therefore, there is an urgent need to address food insecurity by also addressing the known upstream determinants (e.g. policies mitigating climate change, distribution of food, prioritising people and not corporations), to improve sleep health and subsequent health outcomes.

## Supplementary material

For supplementary material accompanying this paper visit https://doi.org/10.1017/jns.2023.18. click here to view supplementary material
