# Supplementary material for: Food insecurity and sleep health by race/ethnicity in the United States
Source: J Nutr Sci. 2023 May 18;12:e59. doi: 10.1017/jns.2023.18 (PMC10214135; doi:10.1017/jns.2023.18)
Supplement: Supplementary file 1 [file S2048679023000186sup001.docx]

# Supplemental Table 1. Age-standardized Sociodemographic by Food Security Scale, National Health Interview Survey, 2013-2018 (N=177,435) ^a^

|  | **Age** | | | **Sex/Gender** | | **Race/Ethnicity** | | | | | **All** |
| --- | --- | --- | --- | --- | --- | --- | --- | --- | --- | --- | --- |
|  | **18-30** | **31-50** | **>50** | **Women** | **Men** | **NH-White** | **NH-Black** | **Hispanic/**  **Latinx** | **NH-Asian** | |  |
| Worried whether food would run out before we got money to buy more | 16.8% | 14.8% | 10.6% | 13.7% | 11.3% | 9.5% | 24.3% | 20.5% | 7.3% | | 12.6% |
| The food we bought just didn’t last, and didn’t have money to get more | 13.8% | 12.3% | 9.4% | 11.9% | 9.7% | 8.0% | 21.9% | 17.7% | 6.3% | | 10.8% |
| We couldn’t afford to eat balanced meals | 12.2% | 10.8% | 8.7% | 10.5% | 8.9% | 7.4% | 18.0% | 16.1% | 6.1% | | 9.7% |
| You or other adults in family ever cut the size of your meals or skip meals because there wasn’t enough money for food? | 32.9% | 35.5% | 34.2% | 34.7% | 33.7% | 36.3% | 36.4% | 29.8% | 22.2% | | 34.2% |
| In the last 30 days, how many days did this happen? | 6.4 (0.03) | 6.0  (0.02) | 5.0  (0.02) | 5.9  (0.02) | 5.3  (0.02) | 4.8  (0.01) | 10.4  (0.05) | 7.0  (0.04) | 2.3  (0.05) | 5.6  (0.01) | |
| Did you ever eat less than you felt you should because there wasn’t enough money for food | 33.9% | 22.5% | 36.2% | 36.0% | 36.2% | 37.2% | 39.9% | 31.7% | 25.6% | | 36.1% |
| Were you ever hungry but didn’t eat because there wasn’t enough money for food? | 20.9% | 22.5% | 20.6% | 20.6% | 21.7% | 22.9% | 22.2% | 17.0% | 14.7% | | 21.1% |
| Did you lose weight because there wasn’t enough money for food? | 11.5% | 12.5% | 13.8% | 12.5% | 13.9% | 13.8% | 14.2% | 11.3% | 10.5% | | 13.1% |
| Did you or other adults in your family ever not eat for a whole day because there wasn’t enough money for food? | 16.5% | 17.5% | 18.4% | 16.6% | 19.6% | 18.6% | 20.5% | 13.7% | 15.7% | | 17.9% |
| How many days did this happen? | 9.1  (0.01) | 10.9  (0.01) | 8.6  (0.01) | 8.9  (0.01) | 9.9  (0.01) | 7.8  (0.01) | 21.2  (0.02) | 10.2  (0.01) | 2.1  (0.01) | | 9.4  (0.01) |

^a^ Note all estimates are weighted for the survey’s complex sampling design. All estimates are age-standardized to the US 2010 population, except for age.

# Supplemental Figure 1. Flow Chart of Participant Selection

Final analytic sample:

NH-White, NH-Black, Hispanic/Latinx, and Asian women and men ≥18 years old meeting eligibility criteria

**(N= 177,435)**

Total NHIS Study Sample, 2013-2018

**(N= 190,113)**

Exclusion Criteria

- Missing data
  - Food Security (n=44)
  - Sleep Duration (n=6,126)
  - Sleep Quality (n=1,643)
  - Race/ethnicity (n=3,464)
- Native American or other race/ethnicity (n= 1,401) due to small sample size
- **Total excluded (n = 12,678)**

# Supplemental Figure 2. Food Security Status by Sex/Gender, National Health Interview Survey, 2013-2018 (N=177,435)

# Supplemental Figure 3. Food Security Status by Sleep Duration, National Health Interview Survey, 2013-2018 (N=177,435)

**Supplemental Figure 4. Food Security Status by Sleep Disturbances**
